# Impact of Sc^3+^-Modified Local Site Symmetries on Er^3+^ Ion Upconversion Luminescence in Y_2_O_3_ Nanoparticles

**DOI:** 10.1021/acs.jpcc.2c00835

**Published:** 2022-07-06

**Authors:** Yuming Wang, Xianli Wang, Yuanbing Mao, James A. Dorman

**Affiliations:** † Cain Department of Chemical Engineering, Louisiana State University, Baton Rouge, Louisiana 70803, United States; ‡ Department of Chemistry, Illinois Institute of Technology, Chicago, Illinois 60616, United States

## Abstract

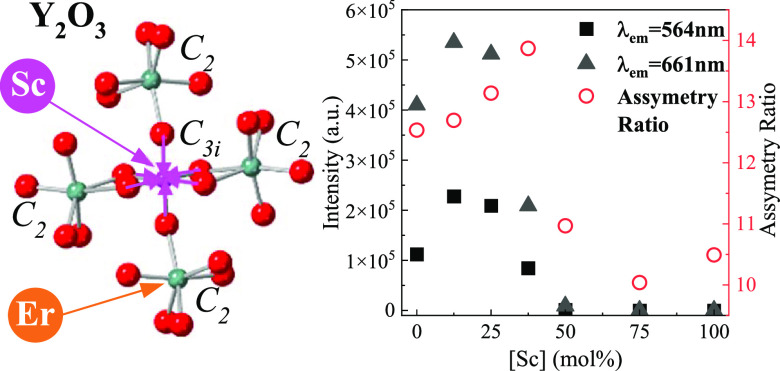

Rare earth (RE) doped yttria sesquioxide has been widely used as host materials for upconversion (UC) phosphors due to their high refractive index, wide band gap, and high melting point. Meanwhile, while fluoride matrices with low phonon cutoff energies exhibit stronger UC emissions, RE-doped oxides exhibit better thermal stability and higher thermal sensitivity when applied as optical temperature sensors. In this work, Sc^3+^ is substituted in RE-doped Y_2_O_3_ lattices to generate smaller cation sites, enhancing the crystal field and modifying the allowed optical transitions. Er^3+^ is used as a photoluminescent probe to study the effect of site position and symmetry on the UC performance. In comparison with the traditional hydrothermal method, Sc^3+^ is successfully incorporated into the Y_2_O_3_ lattice via the co-precipitation/molten salt method without segregating observed. The Judd–Ofelt analysis was applied to determine the local symmetry and efficiency changes. Sc was found to be able to improve the luminescence performances of Er in Y_2–*x*_Sc_*x*_O_3_ (YScO) hosts by adjusting the local symmetry level around the luminescent sites. The local symmetry level was reduced with less than 30 mol % of Sc doping concentration based on the changes in Ω_2_ values. Meanwhile, the YScO oxide was found to significantly improve the luminescence intensity and red-to-green ratio at a lower Yb^3+^ concentration (5 mol %) instead of a higher concentration (20 mol %) commonly used. This was attributed to an increased energy transfer between the closer Yb^3+^–Er^3+^ pairs. Overall, this work allows the spatial occupancy of luminescence centers in the metal oxide host materials to optimize the UC luminescence performance and develop a high-efficiency oxide material for high-temperature applications such as optical thermometry.

## 1 Introduction

In recent years, upconversion (UC) luminescence of rare earth (RE)-doped nanophosphors has drawn considerable interest due to their potential applications in lasers,^1,2^ solar cells,^3−5^ biological fluorescence imaging and detection,^6,7^ IR quantum counters,^8^ and display technologies.^9^ Among the luminescent RE elements, Er^3+^, Ho^3+^, and Tm^3+^ are commonly used as UC activators due to their ladder-like arranged energy levels, long lived excited states, and excellent resonance.^10,11^ Meanwhile, Er^3+^ shows particularly high UC efficiency owing to its similar sequential energy gaps, e.g., ^4^I_11/2_ and ^4^I_15/2_ states and ^4^F_7/2_ and ^4^I_11/2_ states, for sequential IR excitation,^10,11^ which can be enhanced through the incorporation of sensitizers (i.e., Yb^3+^), reducing the possibility of non-radiative relaxation. Yb^3+^ sensitization is commonly used due to its large absorption cross section (9 × 10^–21^ cm^–2^, ^2^F_7/2_ → ^2^F_5/2_) and simple energy-level structure with only one excited state of ^2^F_5/2_, which matches well with those of many RE ions.^12^ Additionally, a number of common dopants have been investigated to improve the luminescence properties, including Li^+^, Sr.^+^, Ca^2+^, Sr^2+^, etc.^13−16^ However, care must be taken to avoid changing the crystal structure (Sr/Ca) or prevent unwanted energy transfer, which leads to enhanced quenching (Li).^17^

Alternatively, the UC efficiency can be improved by modifying the host material to prevent non-radiative relaxation via parasitic surface sites or phonon vibrations.^10^ Fluorides are widely applied as host matrices for UC phosphors, but their low thermal stability limits their application.^11^ Thus, the challenge is to design a material with high chemical and thermal stability and strong UC emissions comparable to fluoride phosphors. Here, pure RE oxide hosts are applied as a host material for UC phosphors due to their high melting points, high refractive indexes, large band gaps, excellent physical and chemical stability, and low phonon energy.^18−23^ Specifically, yttrium oxide (Y_2_O_3_) forms a sesquioxide with *C*_2_ (octahedral) and *C*_3*i*_ (trigonal prismatic) point symmetries.^24^ One unit cell contains 32 cation sites, with 24 of these with *C*_2_ point symmetry and 8 with *C*_3*i*_.^24^ For luminescence applications, the optical emissions are assigned to the electronic transition of the dopant ions in the *C*_2_ sites, while those of the dopants in the *C*_3*i*_ sites are forbidden due to the inversion symmetry. Similar to Y_2_O_3_, Sc_2_O_3_ has the same cubic bixbyite structure with a smaller cation size (Y^3+^-102 pm vs Sc^3+^-74.5 pm), creating smaller dopant sites and an enhanced local crystal field. Meanwhile, it is known that the luminescence performance can be improved if the local structure of the RE site has a lower symmetry level.^25^ As such, the larger RE ions occupying the *C*_2_ sites are expected to prompt higher luminescence intensity of dopant ions. Co-doping the Y_2_O_3_ system with Sc^3+^ allows for manipulating lattice site symmetry to concentrate Er^3+^ in the lower symmetric *C*_2_ sites.

In this work, Y_2–*x*_Sc_*x*_O_3_ (YScO):Er^3+^ (5 mol %) and YScO:Er^3+^ (5 mol %),Yb^3+^ (*y* mol%, *y* = 5, 10, 15, 20) nanoparticles (NPs) were synthesized via a co-precipitation/molten salt synthesis process with varying Sc^3+^ molar ratios. Structural characterization was performed using XRD to affirm the doping homogeneity and crystallinity of the NPs. Next, the optical performance was determined using photoluminescence (PL) spectroscopy, showing that the YScO:Er^3+^ NPs with low Sc concentration (*x* < 0.5) exhibit improved UC intensity. To determine the Sc effect on the UC efficiency, Judd–Ofelt calculations were applied to each same using the PL excitation spectra measured at 77 K.^26^

## 2 Experimental Section

### 2.1 Sample Preparation of Yttrium Scandium Oxide (YScO) NPs

YScO:Er^3+^ and YScO:Er^3+^,Yb^3+^ NPs were synthesized using both the co-precipitation/molten salt method and hydrothermal method.^16,27,28^

YScO:Er^3+^ and YScO:Er^3+^,Yb^3+^ with seven different molar ratios ((mol Sc)/(mol Y + mol Sc) = 0, 0.125, 0.25, 0.375, 0.5, 0.75, and 1) and four different Yb^3+^ concentrations (5, 10, 15, and 20 mol %) were synthesized using a two-step co-precipitation/molten salt method. For example, the Y_1.75_Sc_0.25_O_3_:Er^3+^ (5 mol %),Yb^3+^ (5 mol %) was prepared by dissolving 1.75 mmol of yttrium(III) nitrate hexahydrate (Y(NO_3_)_3_·6H_2_O, Alfa Aesar, 99.9%), 0.25 mmol of scandium(III) chloride hexahydrate (ScCl_3_·6H_2_O, Alfa Aesar, 99.9%), 0.1 mmol of erbium(III) chloride hexahydrate (ErCl_3_·6H_2_O, Aldrich Chemistry, 99.9%), and 0.1 mmol of ytterbium(III) chloride hexahydrate (YbCl_3_·6H_2_O, Aldrich Chemistry, 99.9%) in 50 mL of deionized (DI) water under vigorous magnetic stirring. Next, 50 mL of ammonium hydroxide (NH_4_OH, 28–30%, ACS grade) was added to the above solution dropwise and stirred for 2 h. The precipitate was filtered and washed with DI water several times until the pH of the solution was 7. After washing and filtering, the precipitate was dried overnight at 100 °C. The dried precipitate was then mixed and grounded with a eutectic mixture of NaNO_3_ (high purity grade, VWR, 99.0%) and KNO_3_ (ACS grade, VWR) to form a homogeneous powder. The mixture was heated to 650 °C for 6 h with a heating rate of 10 °C/min. After cooling to room temperature, the resultant powder was washed several times with DI water and dried overnight at 100 °C. Then, the as-synthesized powder samples were annealed at 750 °C for 16 h to obtain YScO:Er^3+^ NPs. A similar synthetic route was employed to prepare the YScO:Er^3+^,Yb^3+^ NPs with varying Sc^3+^ and Yb^3+^ doping concentrations.

The hydrothermal method was also used to synthesize the YScO:Er^3+^ (5 mol %) NPs with five different Sc molar ratios (0, 0.25, 0.5, 0.75, and 1).^28^ Taking Y_2_O_3_:Er^3+^ (5 mol %) as an example, 2 mmol of Y(NO_3_)_3_·6H_2_O and 0.01 mmol of ErCl_3_·6H_2_O were dissolved in 4 mL deionized water followed by the addition of 14 mL of 0.2 M NaOH solution drop by drop under stirring. After stirring for 1 h, the solution was transferred into a 23 mL Teflon autoclave and subsequently sealed and then heated at 180 °C overnight (16 h). The autoclave was allowed to cool naturally, and then the precipitate was washed with deionized water and centrifuged for several times. Afterward, the powder was dried at 90 °C overnight, placed into a porcelain crucible and annealed at 500 °C for 3 h.^28^

### 2.2 Powder X-ray Diffraction (XRD)

The crystal structure was studied by performing powder XRD using a PANalytical X-ray diffractometer operating at 45 kV and 40 mA. The 2θ radial scan was performed using a Cu Kα (λ = 1.54 Å) radiation source from 5 to 70° with a step size of 0.03°. Rietveld refinement was performed on the resultant diffraction patterns using the GSAS II software^29^ for structural verification and phase quantification. Full structural refinement was achieved by performing the procedure outlined in ref (16).

### 2.3 Electron Microscopy

Scanning electron microscopy (SEM) and energy-dispersive X-ray spectroscopy (EDX) were taken using a JEOL JSM-6701F scanning electron microscope operating at 10 kV. The YScO:Er^3+^ (5 mol %) NPs powder was sonicated in an ethanol solution and then dispersed on a carbon tape attached to the SEM stage and dried naturally.

### 2.4 Photoluminescence (PL) Spectroscopy

The downconversion photoluminescence measurements were performed on an Edinburgh FLS1000 PL spectrometer equipped with a PMT detector and a 450 W ozone-free Xenon arc lamp as a light source. The powder samples were placed into a quartz spectrophotometer cell (Starna Cells, Inc). The excitation and emission scans were collected with a bandwidth of 3 nm, a dwell time of 0.5 s, and a step size of 1 nm in the measured range of 350–500 (excitation) and 525–750 nm (emission). Judd–Ofelt calculations were performed based on the excitation measurements in the range of 350–550 nm. For upconversion measurements, an MDL-III-980 laser centered at 980 nm with a power of 2500 mW was employed as the excitation source and emission spectra were recorded from 450–750 nm. The system is not equipped with an integrating sphere, preventing any UC quantum efficiency measurements. The lifetime measurements were performed with a microsecond flash lamp (frequency: 25 Hz, 1–2 μs pulse) over the range of 10 ms with a 2 ms delay time, resulting in a 5 μs detector response (2000 channels). The low-temperature measurements were performed using a LINKAM THMS600 temperature-controlled stage, which was added to the spectrometer setup. Liquid nitrogen was used as the cooling agent.

## 3 Results and Discussion

**Figure 1:**
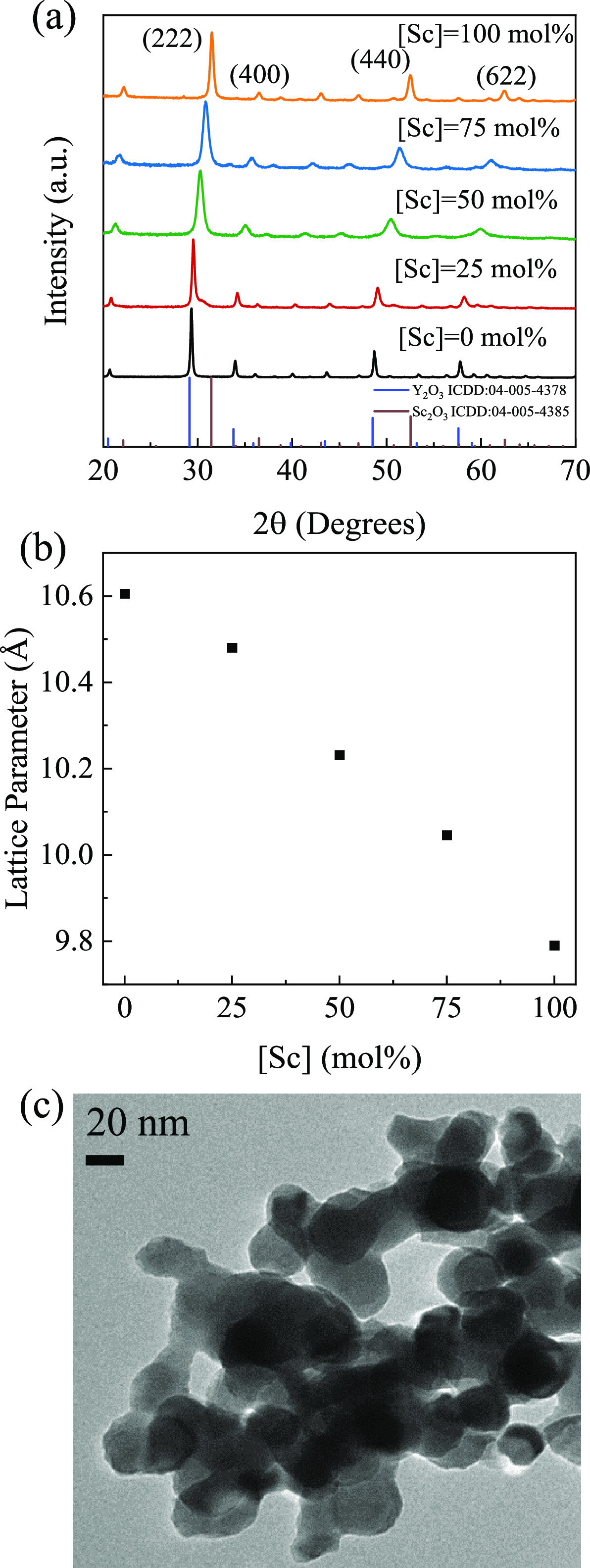
(a) XRD patterns of as-synthesized YScO:Er^3+^ (5 mol %) NPs with Y_2_O_3_ and Sc_2_O_3_ standards using co-precipitation/molten salt synthesis methods. (b) Lattice parameters change as a function of Sc concentration. (c) TEM image of as-synthesized Y_2_O_3_:Er^3+^ (5 mol %).

Y_2_O_3_ and Sc_2_O_3_ are commonly synthesized using the hydrothermal route for phase and NPs shape control.^28,30^ Thus, hydrothermal synthesis was first applied in this work to prepare YScO:Er^3+^ (5 mol %) NPs. As shown in Figure S1 (Supporting Information), Y_2_O_3_:Er^3+^ (5 mol %) and Sc_2_O_3_:Er^3+^ (5 mol %) exhibit highly crystallized phases matching the Y_2_O_3_ (ICDD 05-4378, space group *Ia*3) and Sc_2_O_3_ (ICDD 05-4385, space group *Ia*3) standard phases. No noticeable shift is observed in the Y_2_O_3_ diffraction peaks with Sc^3+^ doping. Instead, a weak diffraction peak at 31.5° appears for the Y_1.5_Sc_0.5_O_3_ host. The peak intensity increases and shifts to match pure Sc_2_O_3_ with increasing Sc^3+^ concentrations, indicating generation of Sc_2_O_3_ segregation instead of a homogeneously mixed YScO lattice. As such, the co-precipitation/molten salt synthesis method was applied to overcome the segregation, as demonstrated for other complex oxides.^25,27,31^ The XRD patterns for the YScO:Er^3+^ (5 mol %) NPs (Figure 1a) are readily indexed to cubic Y_2_O_3_ and Sc_2_O_3_ standards. As expected for mixed metal oxides, the diffraction peaks systematically shift to higher angles with increasing Sc concentration. Furthermore, the lattice parameter decreases linearly from 10.6 (Y_2_O_3_) to 9.8 Å (Sc_2_O_3_), suggesting a homogenous doping of Sc at all concentrations (Figure 1b).

At the same time the particle size decreases proportional to Sc^3+^ concentrations (30.25 nm for Y_2_O_3_ to 17.24 nm for Sc_2_O_3_) based on the Scherrer equation applied to the (220), (400), (440), and (622) peaks. The size matches well with TEM image (Figure 1c). The diffraction patterns were also refined to confirm the crystal structure and particle sizes. All four YScO:Er NPs listed in the Table S1 (Supporting Information) were refined using a cubic bixbyite structure with the *Ia*3 space group, in which Y_2_O_3_:Er^3+^ (5 mol %), Y_1.75_Sc_0.25_O_3_:Er^3+^ (5 mol %) and YScO_3_:Er^3+^ (5 mol %) are refined with the cubic Y_2_O_3_ phase,^32^ while Sc_2_O_3_:Er^3+^ (5 mol %) is refined with the cubic Sc_2_O_3_ phase.^33^ The fits were deemed to be good based on the statistical values (*R*_p_ and *R*_wp_ < 10% and χ^2^ < 10) coupled with visual confirmation (Figure S2). The fitted lattice parameters show good agreement with the calculated values in Figure 1b. Additionally, SEM/EDX elemental mapping were applied to visualize the contribution of each cation in Y_2_O_3_, YScO_3_, and Sc_2_O_3_ NPs (5 mol % Er^3+^, Figures S3–S5). As shown in the figures, no Sc^3+^ or Y^3+^ clustering is present, confirming the NP homogeneity, allowing for further characterization of the optical properties.

**Figure 2:**
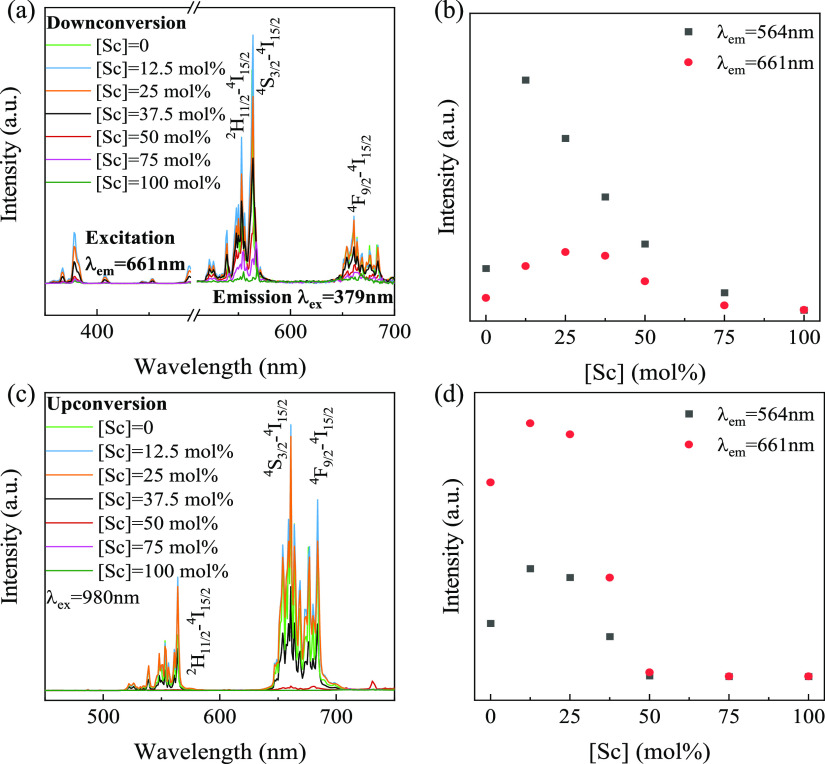
Luminescence performance of YScO:Er^3+^ (5 mol %) NPs with varying Sc concentrations. (a) PL emission spectra excited at 379 nm and excitation spectra with the emission wavelength of 661 nm. (b) Change of PL emission intensities of the peaks at 564 and 661 nm with increasing Sc concentrations. (c) UC spectra excited with a 980 nm laser. (d) Change of UC intensities of the peaks at 564 and 661 nm with increasing Sc concentrations.

DC and UC spectra were collected to determine the luminescence performance of the as-synthesized YScO:Er^3+^ (5 mol %) NPs. Figure 2a shows the DC emission and excitation spectra as a function of Sc^3+^ concentration (measured at room temperature). All YScO:Er^3+^ (5 mol %) NPs exhibit typical Er^3+^ DC emission peaks at 553 nm (^2^H_11/2_ → ^4^I_15/2_), 564 (^4^S_3/2_ → ^4^I_15/2_), and 661 nm (^4^F_9/2_ → ^4^I_15/2_) with the 379 nm excitation.^5^ The DC intensity significantly increases (×5) with Sc^3+^ concentrations up to ∼12 mol % (Figure 2b). Similar enhancement is detected for the UC luminescence (Figure 2c). The maximum UC intensity is observed with 12–25 mol % Sc^3+^ doping, after which the PL nearly completely quenches (Figure 2d). In addition, Figure S6 shows the DC and UC spectra for lower Sc concentration (<12.5 mol %) doped YScO:Er NPs showing increasing intensities that peak at 12.5 mol %. The CIE color coordinates (Figure S7) show no systematic trend with Sc^3+^ concentration in YScO:Er^3+^ (5 mol %) NPs. Overall, the DC and UC spectra improve with lower Sc^3+^ incorporation, enhancing both radiative transitions and is believed to be due to the Sc ion modifying the crystal field around the luminescence center.

**Figure 3:**
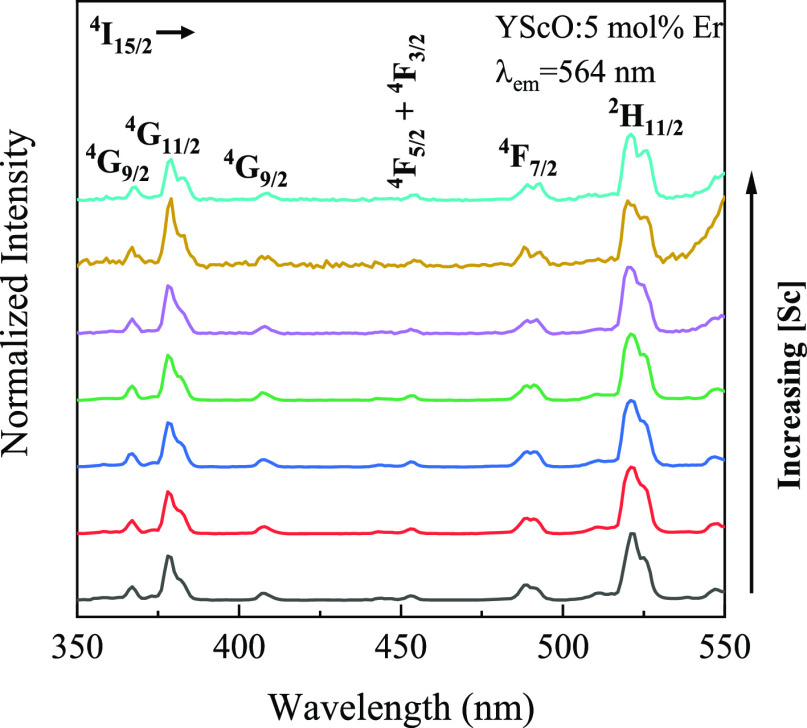
PL excitation spectra with the emission wavelength of 564 nm of YScO:Er^3+^ (5 mol %) NPs with varying Sc concentrations measured at −190 C under a liquid nitrogen environment.

Next, the Judd–Ofelt analysis was applied to quantify the role of Sc^3+^ doping on the luminescence efficiency using the excitation spectra between 350 and 550 nm and monitoring the 564 nm emission peak.^26^ As shown in Figure 3, the characteristic excitation bands of Er^3+^ ions originating from the ^4^I_15/2_ ground state to higher excited states were readily identified.^34^ Six Er^3+^ excitation bands were selected for the evaluation of the Judd–Ofelt parameters, Ω_*t*_ (*t* = 2, 4, 6), for the corresponding Er^3+^ transitions.^26,35,36^ It has been reported that Ω_2_ is closely related to the hypersensitive transitions, which means that larger Ω_2_ values indicate lower local symmetry.^37^ The hypersensitivity of the certain lines in the spectra of RE ions originates from the inhomogeneity of the local crystal environment, with the most striking effect expected for highly polarized, asymmetric environments.^38^ The calculated Judd–Ofelt parameters are shown in Table 1. The values of the Judd–Ofelt parameters for the Y_2_O_3_:Er^3+^ (5 mol %) NPs (Ω_2_ = 6.71 × 10^–20^ cm^2^, Ω_4_ = 2.76 × 10^–20^ cm^2^, Ω_2_ = 1.54 × 10^–20^ cm^2^) are comparable to the literature validating the measurement method.^39^ The Ω_2_ of the YScO:Er^3+^ (5 mol %) particles peaks at 7.31 × 10^–20^ cm^2^ for 37.5 mol % of Sc^3+^ (Y_1.25_Sc_0.75_O_3_:Er^3+^ (5 mol %)), agreeing with the DC/UC concentration-dependent measurements. In addition to Ω_2_, Eu^3+^ was applied as a symmetry probe to replace the Er^3+^ and further determine the local symmetry change around the luminescent ion sites (Figure S8). The magnetic dipole (MD, λ_em_ = 590 nm) and electric dipole (ED, λ_em_ = 611 nm) transitions for Eu^3+^ are commonly used to quantify the local environment change through the asymmetry ratio (*R* = *I*_ED_/*I*_MD_).^25^ The calculated asymmetry ratios are shown in Table S2 and match well with the Judd–Ofelt analysis, indicating that the Sc incorporation changes the local symmetry in the Er^3+^ sites, which proves the hypothesis made from the PL results.^37^

**Table 1:** Judd–Ofelt Parameters Based on PL Excitation of YScO:Er^3+^ (5 mol %) NPs at 563 nm Emission Measured under a Liquid Nitrogen Environment

[Sc] (mol %)	Ω_2_ (× 10^–20^ cm^2^)	Ω_4_ (× 10^–20^ cm^2^)	Ω_6_ (× 10^–20^ cm^2^)
0	6.71	2.76	1.54
12.5	7.09	2.45	1.60
25	7.09	2.18	1.71
37.5	7.31	1.69	1.78
50	5.72	4.50	0.91
75	6.10	4.12	1.63
100	5.42	5.99	1.17

Next, low-temperature (77 K) lifetimes (Figure S9) were measured for the YScO:Er^3+^ NPs and fitted using single exponential decay function to quantify the luminescence efficiency (Table 2).^40^ The measured lifetimes of emissions at 553 nm (^2^H_11/2_ → ^4^I_15/2_) and 564 nm (^4^S_3/2_ → ^4^I_15/2_) peaks with the addition of 12.5 mol % of Sc^3+^ also show a maximum between 12.5 and 25 mol % Sc incorporation, matching the concentration window of seen for the UC and DC intensity measurements. As with the other luminescence measurements, the ^2^H_11/2_ → ^4^I_15/2_ and ^4^S_3/2_ → ^4^I_15/2_ transitions also show a maximum between 12.5 and 25 mol % Sc^3+^ incorporation. For the Y_2_O_3_:Er^3+^ (5 mol %) NPs, the photons needed (*n*) to excite the ^2^H_11/2_ and ^4^S_3/2_ energy levels is expected to be 2.^41^ In the case of this work, the pump power-dependent intensity of the as-synthesized YScO:Er^3+^ (5 mol %) NPs (Figure S10) are not linear. This can be explained by the mechanism of excited state absorption (ESA) and energy transfer UC (ETU). In the case of ESA as the predominant UC mechanism, the number of photons (*n*) is almost independent of pump power.^42^ This non-linear *P*–*I* curve suggests that Sc^3+^ results in a breakdown of the symmetry, negatively impacting the energy transfer between cations. Overall, with less than 30 mol % of Sc^3+^ incorporation in the Y_2_O_3_:Er^3+^ (5 mol %) NPs, the luminescence properties improved due to local symmetry modifications.

**Table 2:** Luminescence Efficiency Based on Judd–Ofelt Calculation and Decay Lifetime of the Transitions ^2^H_11/2_-^4^I_15/2_ (553 nm), ^4^S_3/2_-^4^I_15/2_ (564 nm), and ^4^F_9/2_-^4^I_15/2_ (661 nm) with the 379 nm Excitation of YScO:Er^3+^ (5 mol % ) NPs Measured under a Liquid Nitrogen Environment

[Sc] (mol %)	τ_calc_ (μs)	τ_exp_ (μs)	efficiency	τ_calc_ (μs)	τ_exp_ (μs)	efficiency	τ_calc_ (μs)	τ_exp_ (μs)	efficiency
0	57	32	56%	265	30	11%	226	42	19%
12.5	56	42	75%	256	39	15%	242	36	15%
25	57	39	69%	240	37	15%	254	35	14%
37.5	57	34	59%	232	31	13%	289	29	10%
50	57	25	44%	438	24	5%	173	18	10%
75	56	20	36%	250	20	8%	167	46	28%
100	53	15	28%	338	16	5%	131	10	8%

**Figure 4:**
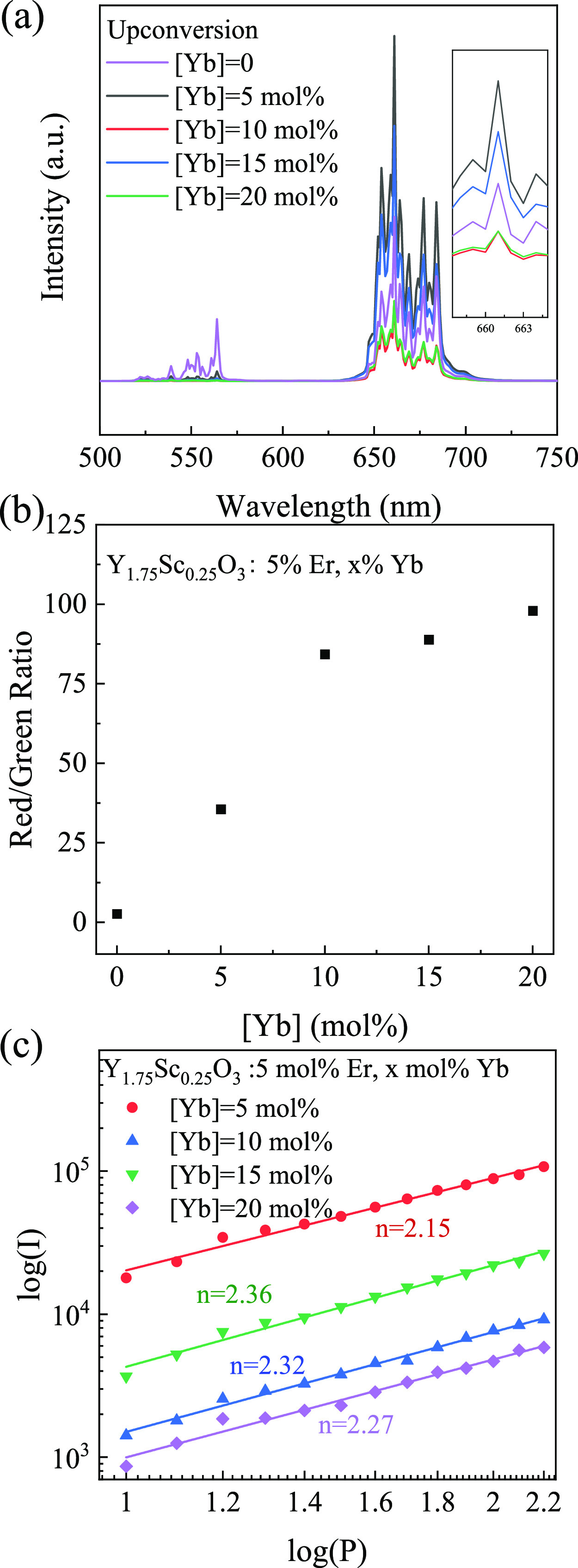
(a) UC spectra, (b) red/green ratio, and (c) pump power dependence of the 661 nm emission intensity of the of Y_1.75_Sc_0.25_O_3_:Er^3+^ (5 mol %),Yb^3+^ (*x* mol%, *x* = 0, 5, 10, 15, 20) NPs.

**Figure 5:**
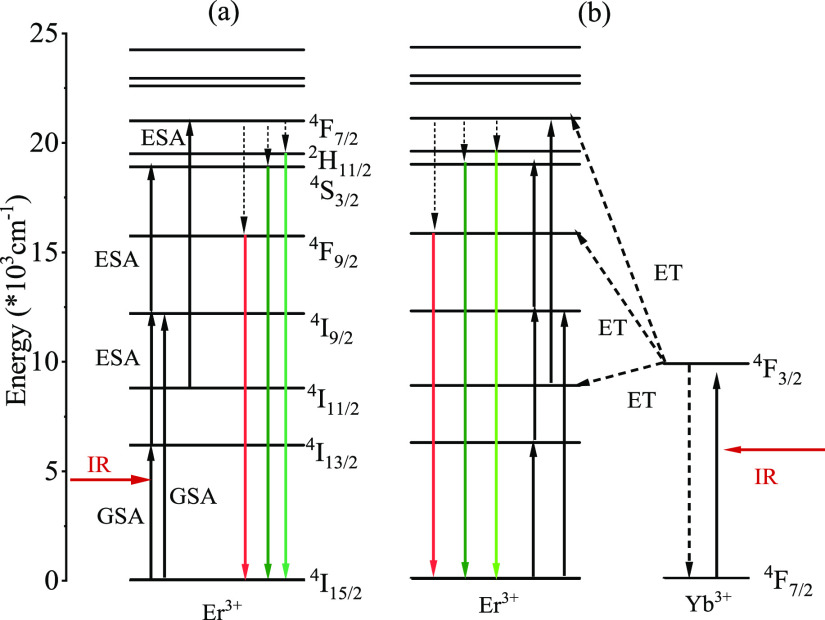
Energy level diagram and energy-transfer mechanism of (a) Er^3+^-doped and (b) Er^3+^/Yb^3+^-co-doped YScO NPs under 980 nm excitation.

As a comparison, the Yb^3+^ ion was co-doped into the YScO:Er^3+^ (5 mol %) NPs as a sensitizer using the previously described co-precipitation/molten salt process (Figure S11). Figure 4 shows the UC spectra of the Y_1.75_Sc_0.25_O_3_:Er^3+^ (5 mol %),Yb^3+^ (0–20 mol %) NPs. Comparison of the UC intensities (insert of Figure 4a) shows that the 5 mol % Yb^3+^ resulted in the highest sensitization for the 661 nm (^4^F_9/2_ → ^4^I_15/2_) emission. Further increasing the Yb doping to 20 mol %^41^ resulted in significant quenching. Additionally, the red to green ratio (*I*_661nm_/*I*_563nm_) increases with increasing Yb^3+^ concentration (Figure 4b) as a result of enhanced Yb^3+^-Er^3+^ energy transfer. This trend is as expected as the distance between RE dopants is inversely proportional to the concentration but also results in enhanced surface quenching (lower intensities). On top of this, it is believed that the larger Yb^3+^ ions (0.87 Å) can expand the unit cell, negating the impact of the smaller Sc^3+^ ions. The power-dependent UC intensity was measured for different Yb^3+^ concentrations (Figure 4c). As opposed to the single Er doped system, the co-doped system exhibited a linear power dependency indicative of efficient Yb^3+^-Er^3+^ energy transfer. Interestingly, the number of required photons (*n*) increases proportionally with Yb^3+^ doping, again suggesting increased energy transfer and quenching due to parasitic surface groups. The UC mechanism is illustrated with the energy level diagram shown in Figure 5. As Er^3+^ is the only dopant in the YScO host, the UC process occurs through ground-state absorption (GSA) followed by excited-state absorption (ESA). The excited electrons relax to a stable state via non-radiative relaxation followed by radiative relaxation (Figure 5a). Since GSA and ESA are both low efficiency excitation processes, Yb^3+^ was introduced into the system due to its large absorption cross section (∼10^–20^ cm^2^).^43,44^ In this case, the GSA and ESA processes are diminished, while tge energy transfer (ET) process becomes the dominant excitation mechanism for Er^3+^, improving the overall UC efficiency (Figure 5b).^45^

Furthermore, to calculate the efficiency of YScO:Er^3+^,Yb^3+^ NPs, the Judd–Ofelt analysis was also applied (Figure S12a and Table 3). The Ω_2_ value increases up to 10 mol % Yb^3+^, indicating further amplification of the hypersensitive transitions (and decreased symmetry) despite the luminescence quenching. To further quantify the luminescence efficiency, lifetimes for the transitions of interest all decreased with increasing Yb^3+^ concentration (Table 4 and Figure S12b–d). Both the experimental and theoretical lifetimes (and efficiency) slightly decreased for the 5 mol % Yb^3+^ sample compared to the samples without Yb^3+^. However, higher dopant concentrations significantly impacted the luminescence efficiency. Based on the slate of PL characterization measurements, the 5 mol % Yb^3+^ was deemed the optimal doping concentration to sensitize the luminescence.

**Table 3:** Judd–Ofelt Parameters Based on PL Excitation of Y_1.75_Sc_0.25_O_3_:Er^3+^ (5 mol %),Yb^3+^ (*x* mol%, *x* = 0, 5, 10, 15, 20) NPs at 563 nm Emission Measured under a Liquid Nitrogen Environment

[Yb] (mol %)	Ω_2_ (× 10^–20^ cm^2^)	Ω_4_ (× 10^–20^ cm^2^)	Ω_6_ (× 10^–20^ cm^2^)
0	7.43	1.87	1.59
5	7.96	2.19	1.43
10	8.12	1.76	1.54
15	7.82	1.65	1.83

**Table 4:** Luminescence Efficiency Based on Judd–Ofelt Calculation and Decay Lifetime of the Transitions ^2^H_11/2_-^4^I_15/2_ (553 nm), ^4^S_3/2_-^4^I_15/2_ (564 nm), and ^4^F_9/2_-^4^I_15/2_ (661 nm) with Excitation at 379 nm of the Y_1.75_Sc_0.25_O_3_:Er^3+^ (5 mol %), Yb^3+^ (*x* mol%, *x* = 0, 5, 10, 15, 20) NPs Measured under a Liquid Nitrogen Environment

	553 nm			563 nm			661 nm		
[Yb] (mol%)	τ_calc_ (μs)	τ_exp_ (μs)	efficiency	τ_calc_ (μs)	τ_exp_ (μs)	efficiency	τ_calc_ (μs)	τ_exp_ (μs)	efficiency
0	56	42	75%	259	39	15%	286	36	13%
5	52	37	71%	287	38	13%	270	30	11%
10	52	19	36%	267	19	7%	300	19	6%
15	54	15	27%	225	13	6%	289	14	5%

Based on the luminescence measurements above, low Sc^3+^ concentrations in the Y_2_O_3_:Er^3+^ NPs improve the Er^3+^ UC and DC intensities by breaking the local symmetries around these sites. By adjusting the size of cation sites with a certain amount of smaller Sc^3+^ ions, the local symmetry around the RE ions is reduced. Interestingly, the phosphors with the highest intensity and luminescence efficiencies had an Y:Sc ratio of ∼3:1, similar to the *C*_2_ and *C*_3*i*_ site amount ratio, suggesting that Sc may preferentially occupy the *C*_3*i*_ sites.

## 4 Conclusions

YScO:Er^3+^ and YScO:Er^3+^,Yb^3+^ NPs were synthesized using co-precipitation and molten-salt synthesis methods. As a comparison to the hydrothermal method, XRD patterns showed a systematic shift of the diffraction peaks and linear change in the lattice parameters with increasing Sc concentrations, indicating that Sc can be incorporated into Y_2_O_3_ lattices and form homogeneous nanocrystals. Sc incorporation was able to improve the overall luminescence performances of Er in the YScO host. Specifically, UC and DC spectra showed that less than 30% of Sc^3+^ incorporation significantly improved the luminescence intensities, in which Y_1.75_Sc_0.25_O_3_:Er^3+^ (5 mol %) NPs exhibited the strongest UC and DC emissions. Furthermore, the comparison between theoretical (Judd–Ofelt analysis) and experimental (lifetimes measurements) methods were used to determine the luminescence efficiency. The calculated Judd–Ofelt parameter Ω_2_ showed that Sc^3+^ substitution of Y^3+^ in Y_2_O_3_ improved the local asymmetry level around the Er^3+^ sites, while Ω_2_ increased with less than 50 mol % of Sc^3+^ and reduced with more Sc^3+^ incorporation. A similar trend was obtained in the fitted lifetime and calculated efficiency, showing that Y_1.75_Sc_0.25_O_3_:Er^3+^ (5 mol %) NPs had the longest lifetime and the highest efficiency and less than 30 mol % of Sc^3+^ is the concentration window, which improved the luminescence performance. Next, Yb^3+^ was introduced into YScO:Er^3+^ as a sensitizer to further optimize the luminescence performance. It was found that Sc^3+^ substitution significantly reduced the Yb^3+^ concentration to a 1:1 Yb^3+^:Er^3+^ ratio. Meanwhile, Sc^3+^ substitution showed the same effect on the YScO:Er^3+^,Yb^3+^ NPs as to the YScO:Er^3+^ NPs where the Y_1.75_Sc_0.25_O_3_:Er^3+^ (5 mol %),Yb^3+^ (5 mol %) NPs exhibited the highest UC and DC luminescence intensities and overall efficiency. Overall, this work offers an idea to spatially control the doping of luminescent RE ions into more asymmetric cation sites to obtain a better luminescence performance, which is required for many applications such as solid-state phosphors, optical thermometers, and luminescence probes.

## Supporting Information Available

Detailed Judd–Ofelt analysis process, XRD patterns of hydrothermal synthesis, Rietveld refinement and refined crystallographic/structural parameters from GSAS II, SEM images, EDX elemental mapping, decay lifetime, power-dependent emission intensity, and CIE 1931 color coordination (PDF)
