# Supplementary material for: Impact of Sc3+-Modified Local Site Symmetries on Er3+ Ion Upconversion Luminescence in Y2O3 Nanoparticles
Source: J Phys Chem C Nanomater Interfaces. 2022 Jul 6;126(28):11715–22. doi: 10.1021/acs.jpcc.2c00835 (PMC9327094; doi:10.1021/acs.jpcc.2c00835)
Supplement: Supplementary file 1 — jp2c00835_si_001.pdf [file jp2c00835_si_001.pdf]

Electronic Supplementary Information:

Impact of Sc^{3+} modified Local Site Symmetries on Er^{3+} Ion Upconversion Luminescence in Y_2O_3 Nanoparticles

Yuming Wang, [a] *Xianli Wang,* [b] *Yuanbing Mao,* [b] *and James A. Dorman* [a,*]

[a] Cain Department of Chemical Engineering, Louisiana State University, Baton Rouge, Louisiana 70803, United States

[b] Department of Chemistry, Illinois Institute of Technology, Chicago, Illinois 60616, United States

**Corresponding author,* jamesdorman@lsu.edu

The Judd-Ofelt analysis is based on assuming the states are completely degenerate in angular momentum, and the energy denominators are equal. The calculated oscillator strength can be written as

$$f_{cal}(aJ, bJ') = \frac{8\pi^2 mc}{3h\lambda(2J+1)e^2n^2}[\chi_{ED}S_{ED}(aJ, bJ') + \chi_{MD}S_{MD}(aJ, bJ')]$$

where χ is local field corrections, for electric dipole transition $\chi_{ED} = \frac{n(n^2+2)^2}{9}$ and for magnetic dipole transition $\chi_{MD} = n^3$. SED and SMD are the line strength of electric dipole transition and magnetic dipole transition, respectively, which are expressed as

$$S_{ED}(aJ, bJ') = e^2 \sum_{\lambda=2,4,6} \Omega_\lambda \left|< 4f^N aJ \middle\| U^\lambda \middle\| 4f^N bJ' >\right|^2$$

$$S_{MD}(aJ, bJ') = \frac{e^2h^2}{4m^2c^2}\left|\langle 4f^N aJ \middle\| \vec{L} + 2\vec{S} \middle\| 4f^N bJ' \rangle\right|^2$$

where U^λ are the irreducible tensor forms of the dipole operator,[1-2] n is the refractive index of the solid, λ is the mean wavelength of the transition, h is Planck's constant, e is the electron charge, m is the mass and c is the speed of light.

Theoretically, it is possible to calculate the Judd-Ofelt parameter, but this requires accurate values for the radial integrals and odd-order crystal field component, which are not known to a high enough degree of precision. Instead, the Judd-Ofelt parameter can be treated as a set of phenomenological parameters to be determined from fitting experimental absorption measurements determined in

$$f_m = \frac{4\varepsilon mc^2}{e^2\lambda^2}\int \sigma_{abs}(\lambda)d\,\lambda = \frac{4\varepsilon mc^2}{e^2\lambda^2}(2.303 \times \int \log(\frac{I_0}{I}))$$

Where $\sigma_{abs}(\lambda)$ is the wavelength dependent absorption cross section.

Once the Judd-Ofelt parameters are determined, they can be used to calculate transition probabilities ($A(J;J')$) of all excited states from the equation,

$$A(J';J) = \frac{64\pi^4e^2}{3h(2J'+1)\bar{\lambda}^3}\left[n(\frac{n^2+2}{3})^2S_{ED} + n^2S_{MD}\right]$$

where S_{ED} and S_{MD} are the electric and magnetic dipole line strength, respectively. Using the transition probability, the radiative lifetime τ_r and the branching ratio β can be calculated as

$$\frac{1}{\tau_r} = \sum_J A(J';J)$$

The luminescence efficiency is calculated based on the ratio of the fitted lifetime from experimental results to the radiative lifetime τ_r. The calculation based on the equations mentioned

above were processed in Excel, and matrix calculations for determining Judd-Ofelt parameters were performed using MATLAB.

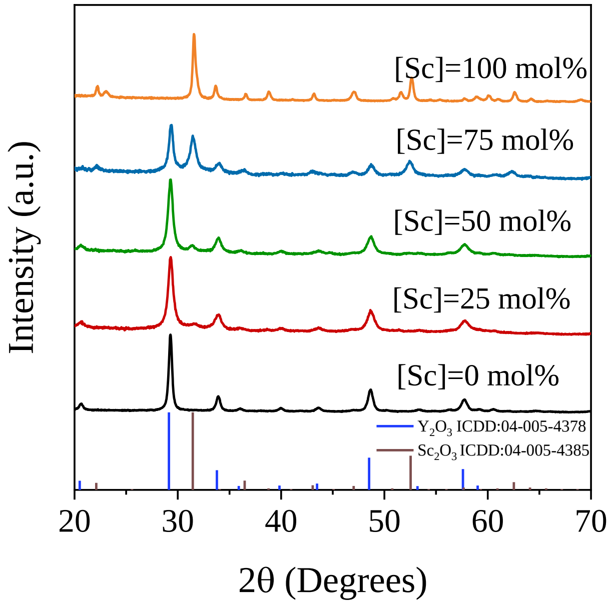

Figure S1. XRD patterns of the as-synthesized YScO:Er^{3+} (5 mol%) NPs with Y_2O_3 and Sc_2O_3 standards using the hydrothermal synthesis.

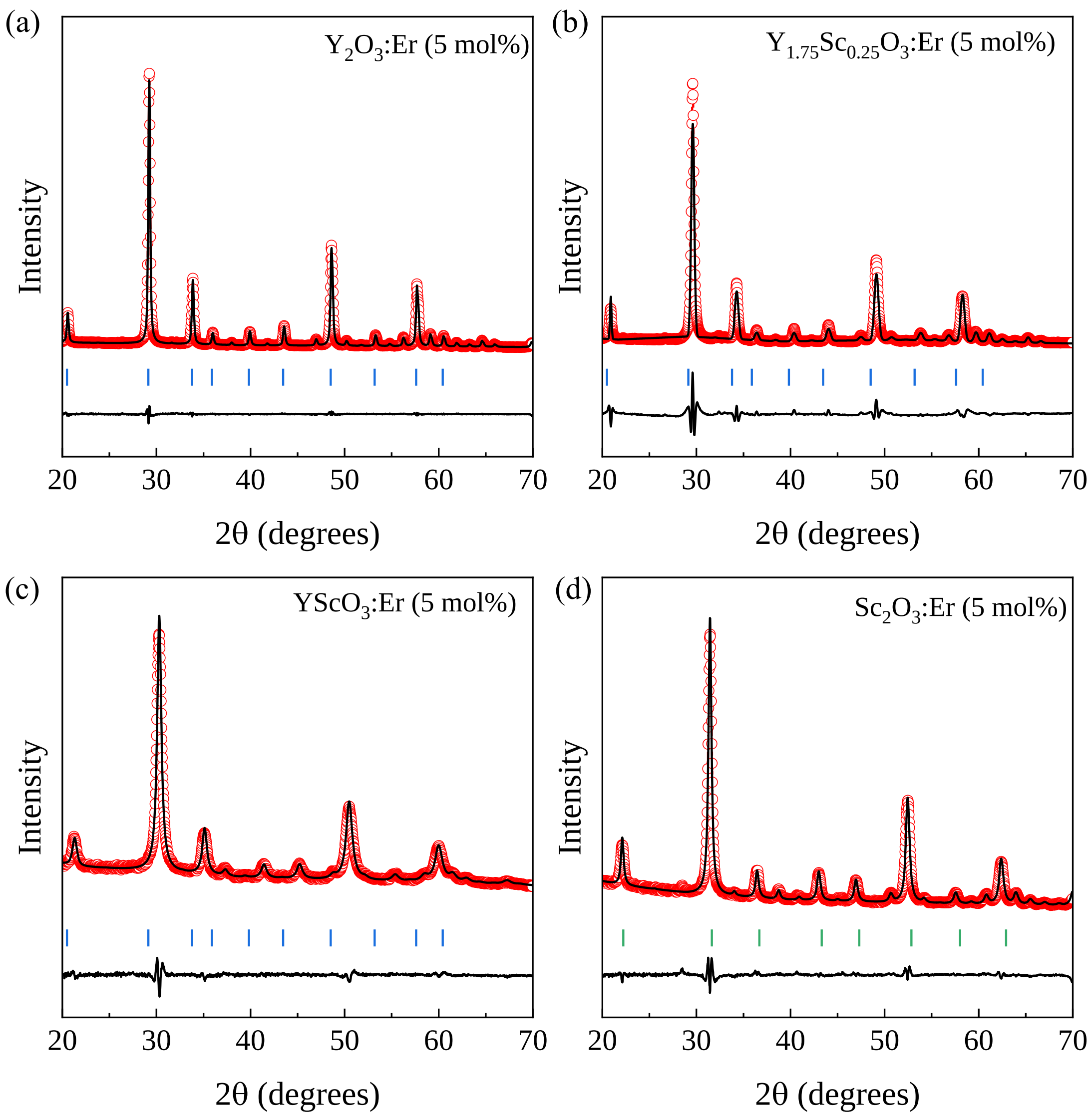

Figure S2. XRD pattern and Rietveld refinement for (a) $Y_2O_3:Er^{3+}$ (5 mol%), (b) $Y_{1.75}Sc_{0.25}O_3:Er^{3+}$ (5 mol%) (c) $YScO_3:Er^{3+}$ (5 mol%) and (d) $Sc_2O_3:Er^{3+}$ (5 mol%) The upper symbols illustrate the observed data (circles) and the calculated pattern (solid line). The vertical markers show calculated positions of Bragg reflections for Y_2O_3 (Blue) and Sc_2O_3 (Green), respectively. The lower curve represents the difference between observed and calculated intensities.

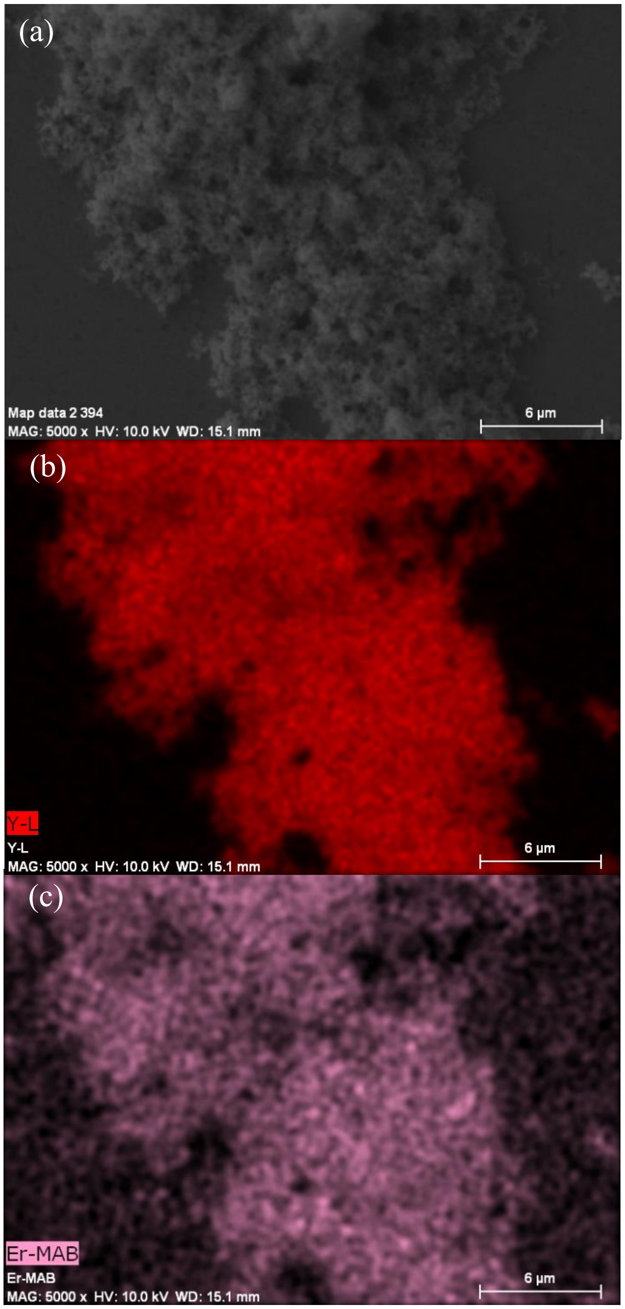

Figure. S3 (a) SEM image and EDX elemental mapping of (b) Y and (c) Er on the as-synthesized Y_2O_3:Er^{3+} (5 mol%) NPs.

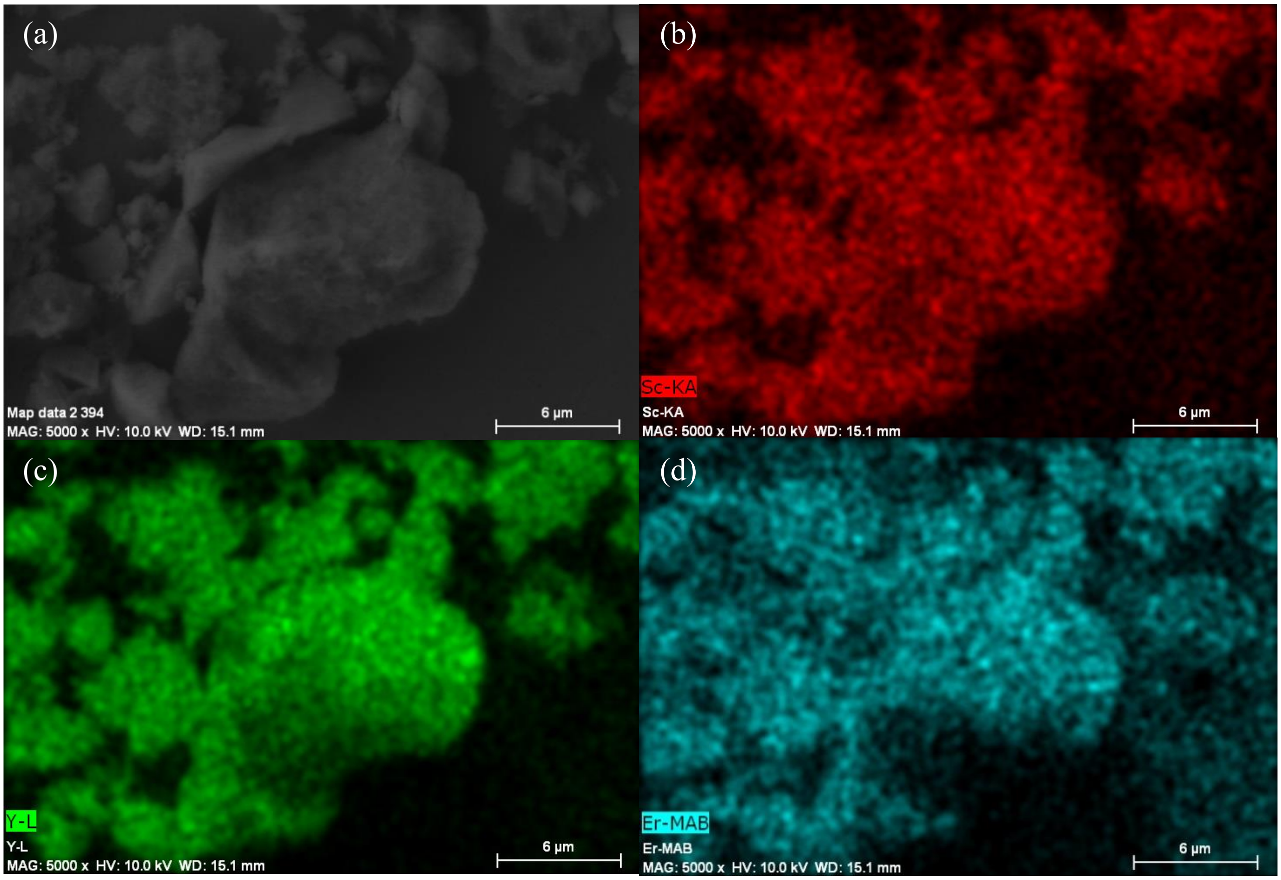

Figure. S4 (a) SEM image and EDX elemental mapping of (b) Sc, (c) Y and (d) Er on the as-synthesized $YScO_3:Er^{3+}$ (5 mol%) NPs.

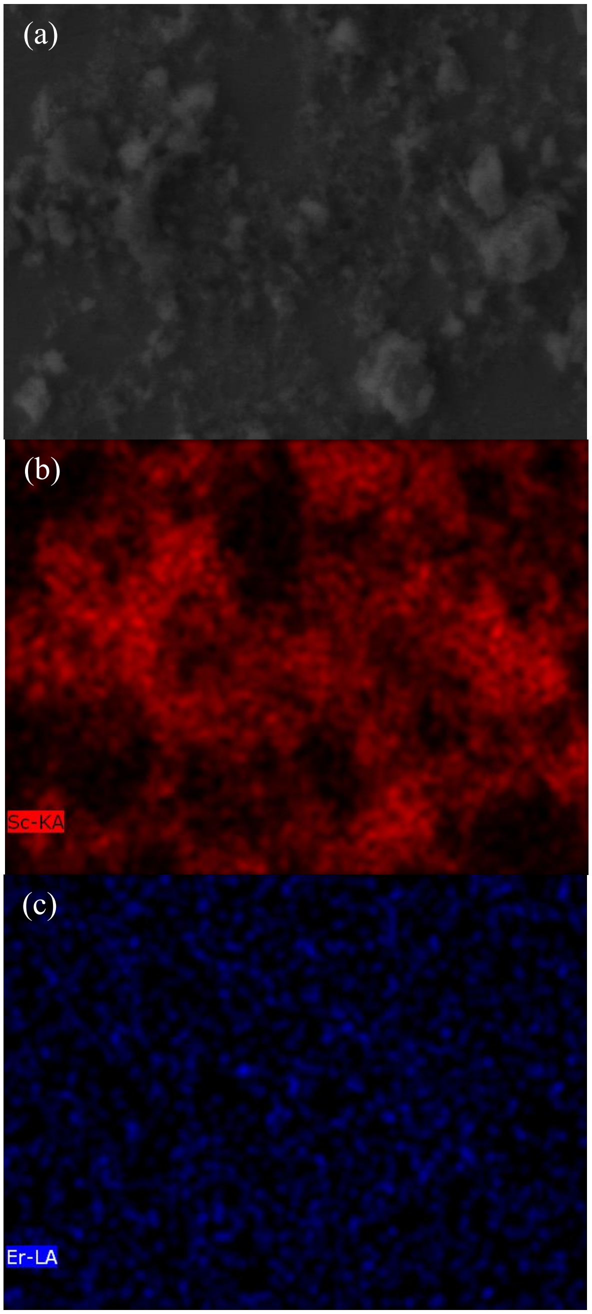

Figure. S5 (a) SEM image and EDX elemental mapping of (b) Sc and (c) Er on the as-synthesized Sc_2O_3:Er^{3+} (5 mol%) NPs.

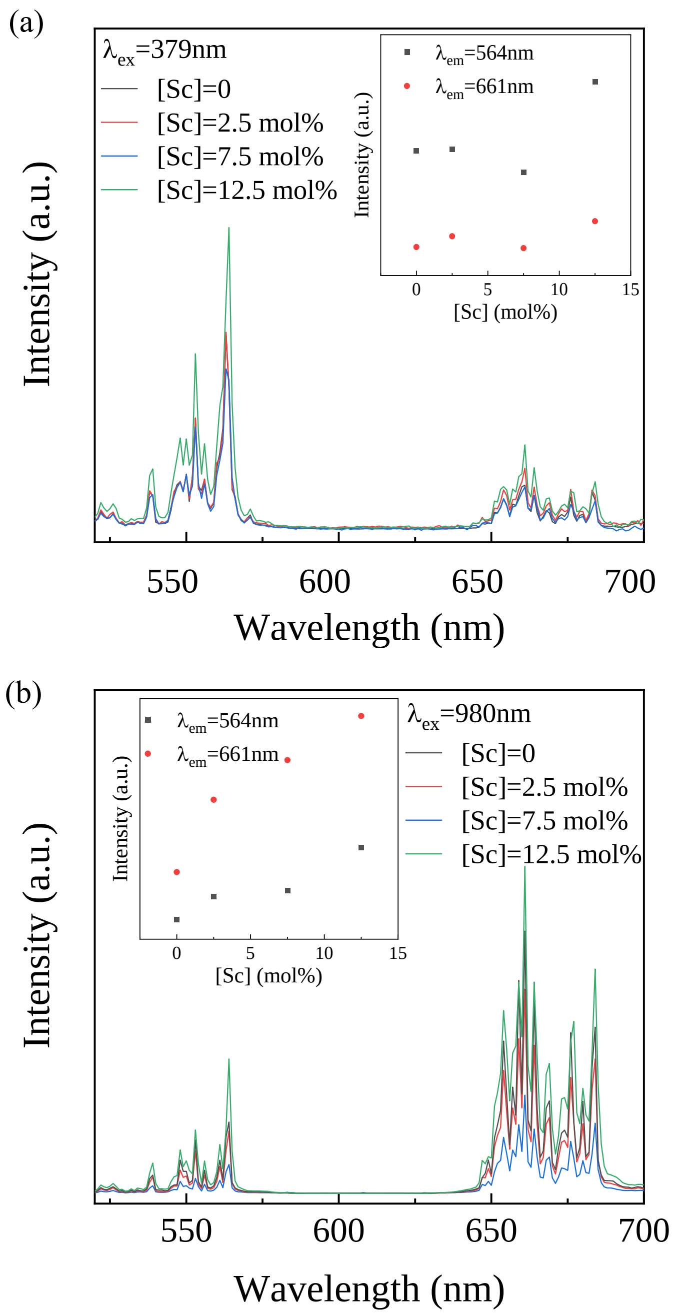

Figure S6. (a) PL DC spectra with 370 nm excitation and (b) UC spectra with 980 nm excitation of YScO:Er^{3+} (5 mol%) NPs with varying Sc concentrations (<12.5 mol%). The inserts show the intensity change with increasing Sc concentrations.

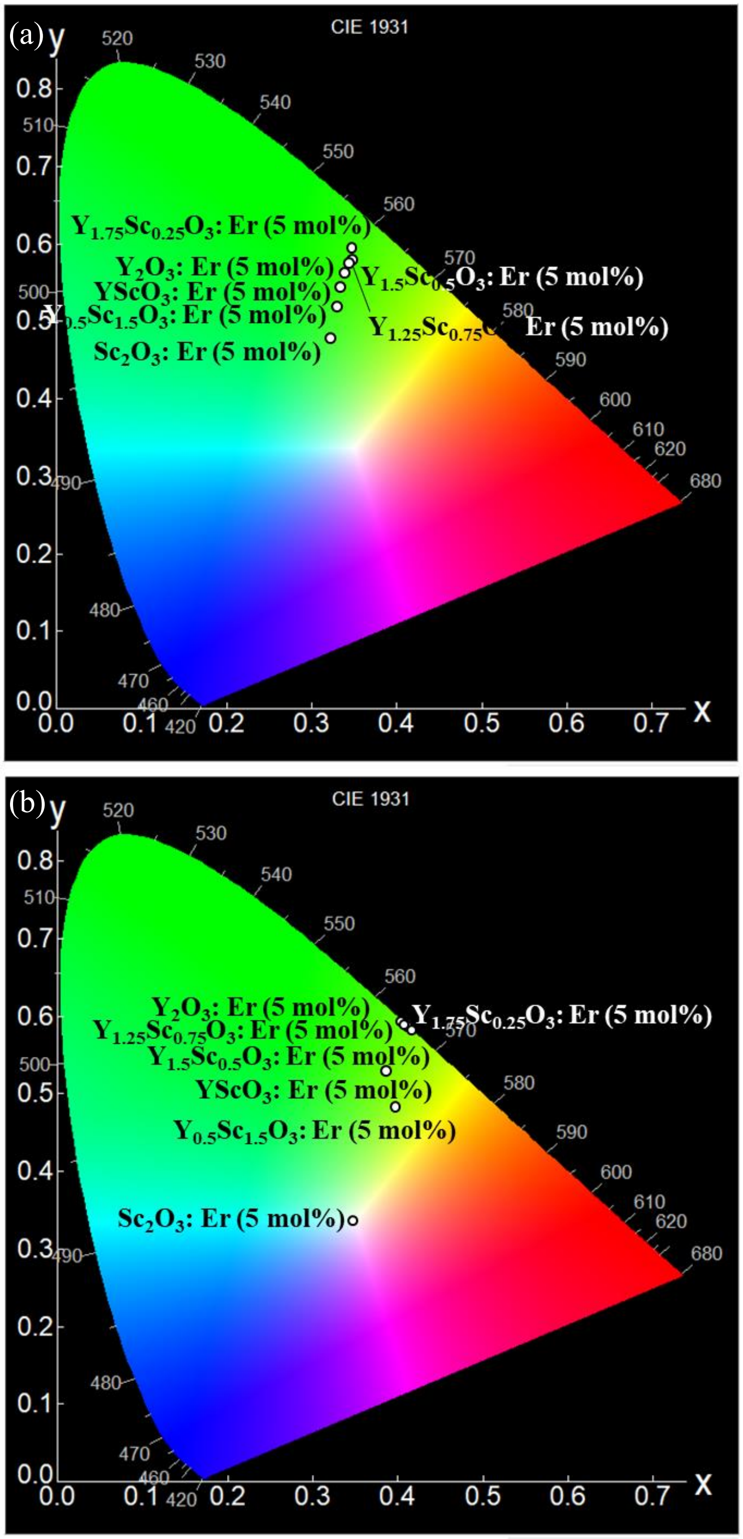

Figure S7. CIE 1931 color coordination of (a) upconversion and (b) downconversion of YScO:Er^{3+} (5 mol%).

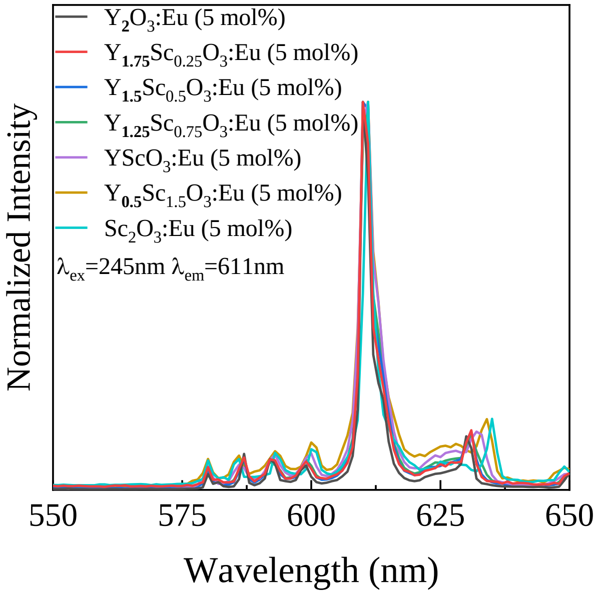

Figure S8. Normalized PL DC spectra of YScO:Eu^{3+} (5 mol%) in which Eu was used as a symmetry probe replacing Er ions to quantify the symmetry change around luminescent ion sites

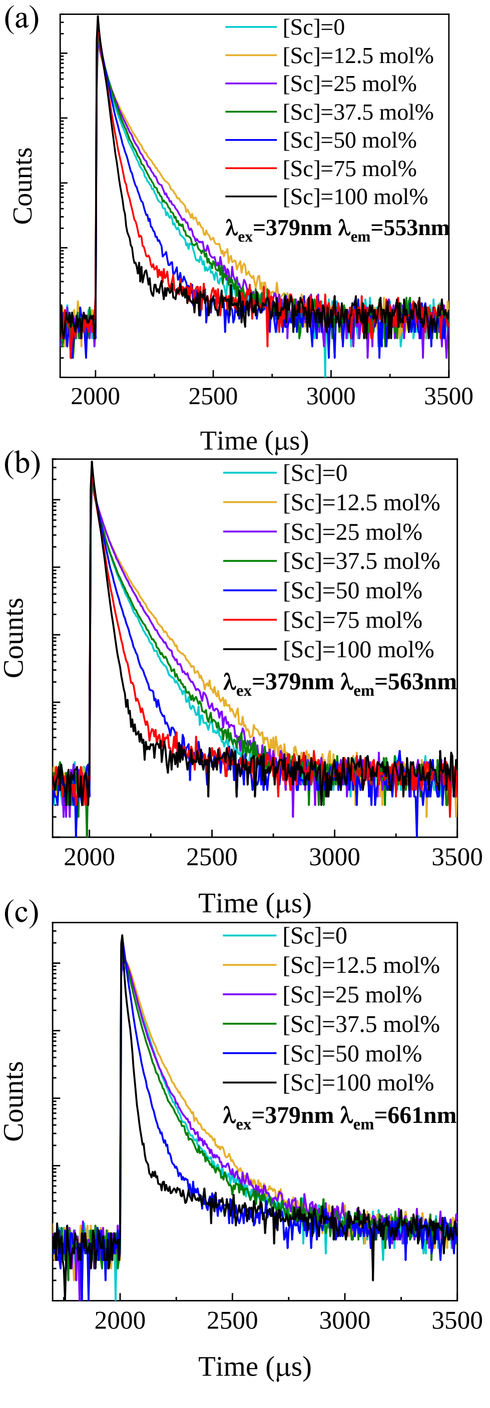

Figure S9 Decay lifetime curves of the (a) $^2H_{11/2} \rightarrow {}^4I_{15/2}$ (553 nm), (b) $^4S_{3/2} \rightarrow {}^4I_{15/2}$ (563 nm), and (c) $^4F_{9/2} \rightarrow {}^4I_{15/2}$ (661 nm) transitions with 379 nm excitation of the YScO:Er^{3+} (5 mol%) NPs measured at -190 °C under liquid nitrogen environment.

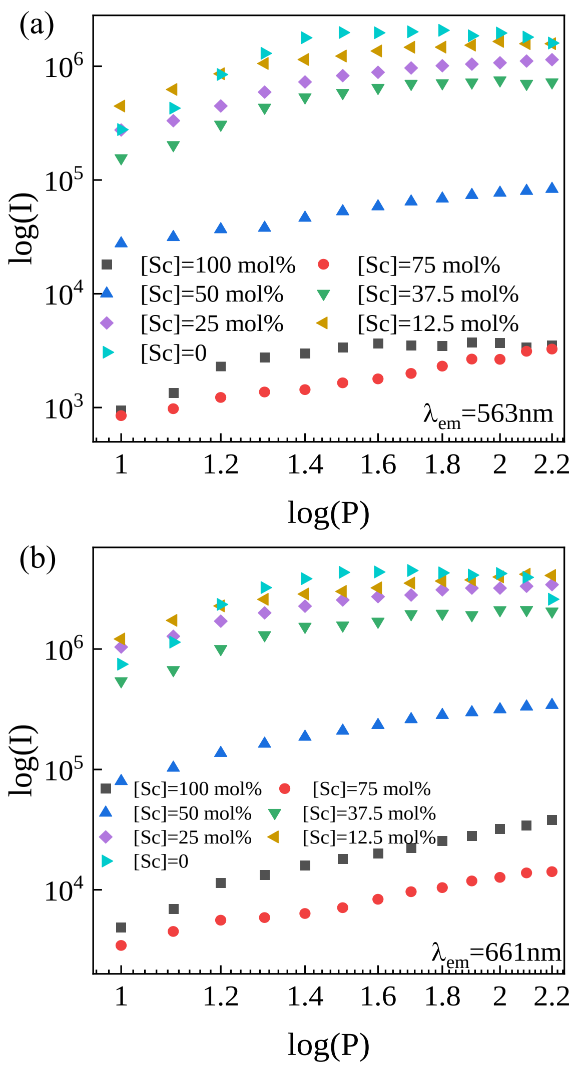

Figure S10 The pump power dependence of the emission intensity at (a) 563 nm and (b) 661nm of the YScO:Er^{3+} (5 mol%) NPs to study the effect of Sc concentration and energy coupling on luminescence.

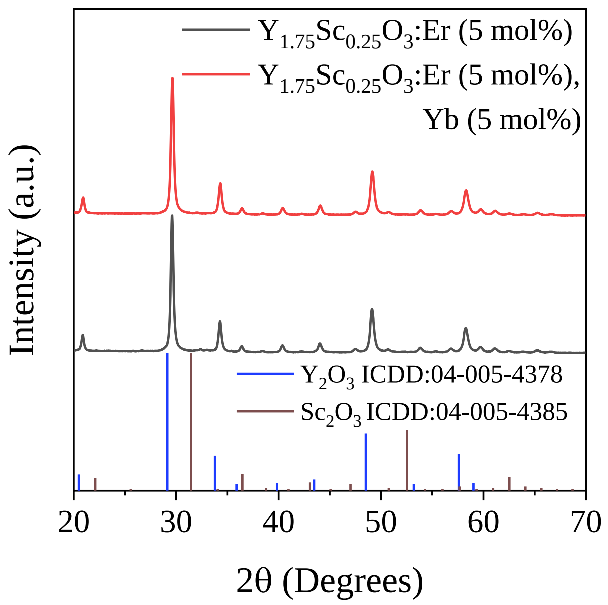

Figure S11. XRD patterns of the as-synthesized $Y_{1.75}Sc_{0.25}O_3:Er^{3+}$ (5 mol%) and $Y_{1.75}Sc_{0.25}O_3:Er^{3+}$ (5 mol%), Yb^{3+} (5 mol%) NPs with Y_2O_3 and Sc_2O_3 standards using the co-precipitation/molten salt synthesis methods.

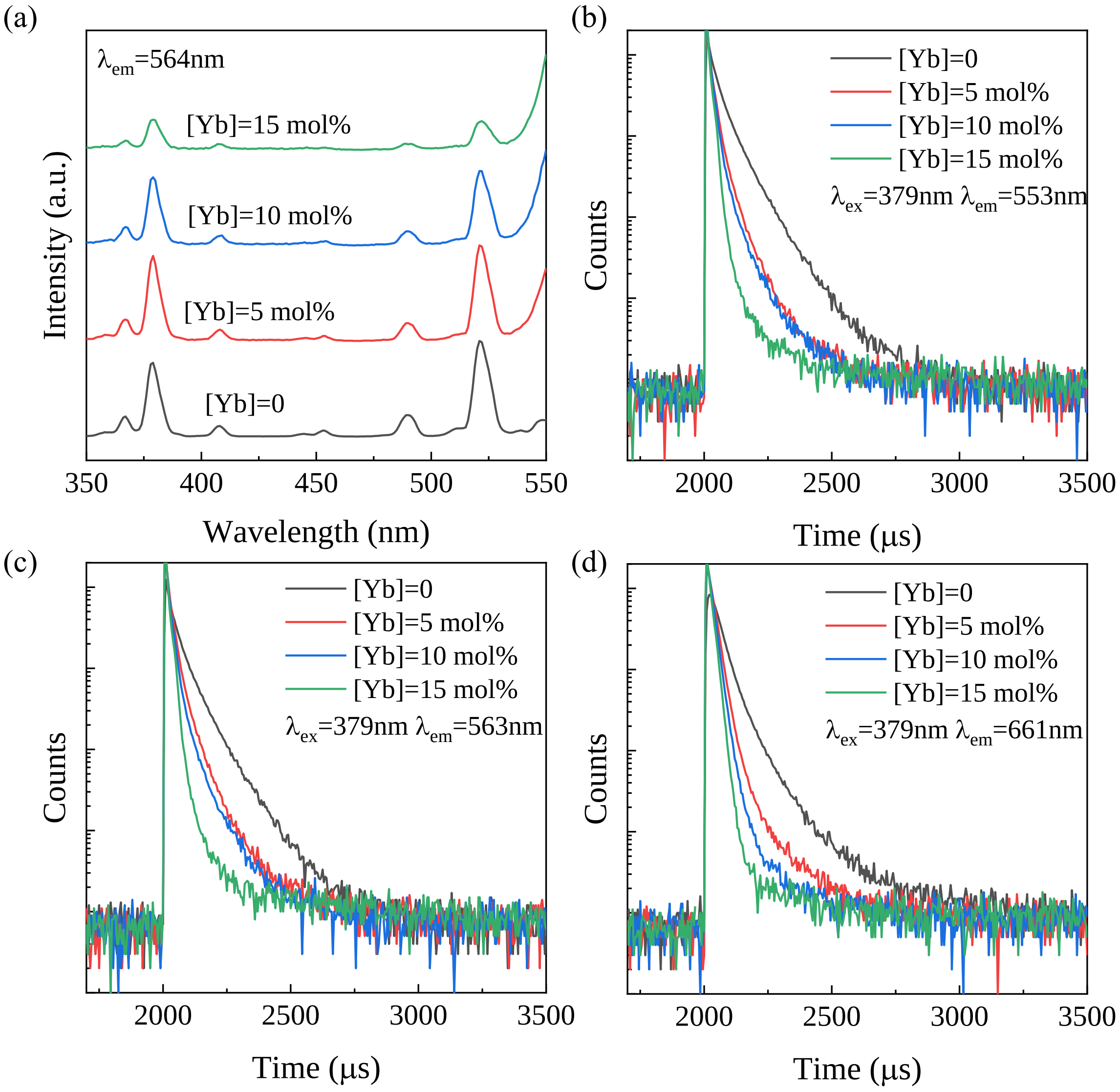

Figure S12. (a) PL excitation spectra with the emission wavelength at 564 nm and decay lifetime curves of the (b) $^2H_{11/2} \rightarrow {}^4I_{15/2}$ (553n m), (c) $^4S_{3/2} \rightarrow {}^4I_{15/2}$ (563 nm), and (d) $^4F_{9/2} \rightarrow {}^4I_{15/2}$ (661 nm) transitions with 379 nm excitation of the $Y_{1.75}Sc_{0.25}O_3$:Er^{3+} (5 mol%), Yb (x mol%, x = 0, 5, 10, 15, and 20) NPs measured at -190°C under liquid nitrogen environment.

Table S1. Crystallographic Data and Refined Lattice Parameters for YScO:Er^{3+} (5 mol%) NPs with varying Sc concentrations based on X-ray Diffraction Data

Parameters	[Sc]=0	[Sc]=12.5%	[Sc]=50%	[Sc]=100%
Wavelength (Å)	1.541			
Temperature (K)	300			
2θ range (°)	20-70			
space group	Ia-3 (No. 206)	Ia-3 (No. 206)	Ia-3 (No. 206)	Ia-3 (No. 206)
Z	1.0			
R_p, R_{wp}(%)	3.06, 2.85	7.34, 7.30	3.06, 2.56	4.33, 4.14
χ^2	3.08	8.51	2.41	2.85
Lattice Parameters				
a,b,c (Å)	10.599(2)	10.510(5)	10.232(9)	9.880(4)
α,β,γ (°)	90	90	90	90
V (Å^3)	1190.989	1161.102	1071.535	964.544
Y1/Sc1 (x,y,z)	0.9687, 0, 1/4	0.9708, 0, 1/4	0.9716, 0, 1/4	0.9571, 0, 1/4
Y2/Sc2 (x,y,z)	1/4, 1/4, 1/4	1/4, 1/4 ,1/4	1/4, 1/4, 1/4	1/4, 1/4, 1/4
O (x,y,z)	0.3923, 0.1539, 0.3807	0.3890, 0.1575, 0.3756	0.3862, 0.1548, 0.3828	0.3908, 0.1558, 0.3799

Table S2. Asymmetry ration based on the intensity ratio of MD and ED transitions of Eu^{3+} in YScO host with varying Sc concentrations.

[Sc](mol%)	I_{ED}/I_{MD}
0	12.53
12.5	12.69
25	13.14
37.5	13.87
50	10.97
75	10.04
100	10.49

REFERENCES

1. Weber, M. J., Probabilities for Radiative and Nonradiative Decay of Er^{3+} in LaF_3. *Physical Review* **1967**, *157*, 262.
2. Weber, M., Radiative and Multiphonon Relaxation of Rare-Earth Ions in Y_2O_3. *Physical Review* **1968**, *171*, 283.
